# A new genus and four new species in the /Psathyrella s.l. clade from China

**DOI:** 10.3897/mycokeys.80.65123

**Published:** 2021-05-26

**Authors:** Tolgor Bau, Jun-Qing Yan

**Affiliations:** 1 Key Laboratory of Edible Fungal Resources and Utilization (North), Ministry of Agriculture and Rural Affairs, Jilin Agricultural University, Changchun 130118, China Jilin Agricultural University Changchun China; 2 Jiangxi Key Laboratory for Conservation and Utilization of Fungal Resources, Jiangxi Agricultural University, Nanchang, Jiangxi 330045, China Jiangxi Agricultural University Nanchang China

**Keywords:** Agaricales, Basidiomycete, four new taxa, Psathyrellaceae, taxonomy

## Abstract

Based on traditional morphological and phylogenetic analyses (ITS, LSU, *tef-1α* and *β-tub*) of psathyrelloid specimens collected from China, four new species are here described: *Heteropsathyrella macrocystidia*, *Psathyrella amygdalinospora*, *P. piluliformoides*, and *P. truncatisporoides*. *H. macrocystidia* forms a distinct lineage and groups together with *Cystoagaricus*, *Kauffmania*, and *Typhrasa* in the /Psathyrella s.l. clade, based on the Maximum Likelihood and Bayesian analyses. Thus, the monospecific genus *Heteropsathyrella* gen. nov. is introduced for the single species. Detailed descriptions, colour photos, and illustrations are presented in this paper.

## Introduction

*Psathyrella* (Fr.) Quél. is characterized by usually fragile basidiomata, a hygrophanous pileus, brown to black-brown spore prints, always present cheilocystidia and basidiospores fading to greyish in concentrated sulphuric acid (H_2_SO_4_) (Kits van Waveren 1985; Örstadius et al. 2015). There are records of more than 1000 names, including synonyms and subspecies, since Fries established the tribe *Psathyrella* under *Agaricus* L. (Fries 1838; Smith 1972; Kits van Waveren 1985; Örstadius and Knudsen 2012). This group has been classified in the Coprinaceae Roze ex Overeem subfamily Psathyrelloideae (Hawksworth et al. 1983; Hawksworth et al. 1995; Kirk et al. 2001) and then incorporated into Psathyrellaceae Vilgalys, Moncalvo & Redhead, based on the study of Redhead et al. (2001). Further studies found that *Psathyrella* is polyphyletic and /Psathyrella s.l. was limited by Örstadius et al. (2015). /Psathyrella s.l. consists of five major supported clades: /*Coprinellus*, /*Cystoagaricus*, /*Kauffmania*, /*Psathyrella* s.s., and /*Typhrasa*. Each major clade represents a genus. Hence, *Kauffmania* Örstadius & E. Larss. and *Typhrasa* Örstadius & E. Larss. were established, C*ystoagaricus* Singer emend. Örstadius & E. Larss. was redefined (Örstadius et al. 2015). Most species in *Cystoagaricus*, *Kauffmania*, and *Typhrasa* were incorporated from *Psathyrella*. Species with cap surface breaking up into dark fibrils or scales were classified into *Cystoagaricus.* Species having rostrate hymenial cystidia with oily drops were classified into *Typhrasa*. *P. larga* (Kauffman) A.H. Sm., which has large basidiomata, scanty veil, and pale spores, was classified into *Kauffmania*.

As a part of the study of Chinese psathyrelloid species, four new species were discovered, during our investigations in temperate and subtropical regions of China from 2016–2019. Among them was a new species morphologically similar to *Psathyrella* but phylogenetically distinguished from it, and which formed a separate lineage. We recognize this new taxon as a new genus based on traditional morphological and phylogenetic analyses. In this paper, detailed information on the new taxa is presented.

## Materials and methods

### Morphological studies

Macroscopic characteristics of fresh specimens were recorded. Colour codes followed Kornerup and Wanscher (1978). Thirty basidiospores, cystidia, and basidia were measured under a microscope in water and 5% aqueous KOH for each specimen. The measurements and Q values are given as (a)b–c(d), in which “a” is the lowest value, “b–c” covers a minimum of 90% of the values and “d” is the highest value. “Q” stands for the ratio of length to width of a spore (Bas 1969; Yu et al. 2020). Photographs of some microscopic characteristics are shown in Suppl. material 1: Figure S1. Specimens were deposited in the Herbarium of Mycology, Jilin Agricultural University (**HMJAU**).

### DNA extraction and sequencing

DNA was extracted from dried specimens with the NuClean Plant Genomic DNA kit (CWBIO, China). Four regions (ITS, LSU, *tef-1α* and *β-tub*) were amplified for the study, which using ITS1/ITS4 (White et al. 1990), LR0R/LR7 (Hopple and Vilgalys 1999), EF983F/EF2218R (Örstadius et al. 2015), and B36f/B12r (Nagy et al. 2011), respectively. PCR was performed using a touchdown program for all regions as follows: 5 min at 95 °C; 1 min at 95 °C; 30 s at 65 °C (add -1 °C per cycle); and 1 min at 72 °C for a cycle of 15 times; 1 min at 95 °C; 30 s at 50 °C; and 1 min at 72 °C for a cycle of 20 times; and 10 min at 72 °C (Yan and Bau 2018b). DNA sequencing was performed by Qing Ke Biotechnology Co., Ltd. (Wuhan City, China), using primers listed above.

### Data analyses

ITS1+5.8S+ITS2 sequences of four new species were tested with BLAST in GenBank, species sharing over 95% similarity are selected. Based on the BLAST results, morphological similarities and then compared to the research of Örstadius et al. (2015) and Yan and Bau (2018a). Totally, 176 sequences of 46 taxa, including 4 regions (ITS, LSU, *tef-1α*, and *β-tub*) which divided into 7 partitions (ITS, LSU, Tef 1^st^, Tef 2^nd^, Tef 3^rd^, Tub 1^st^, and Tub 2^nd^) were downloaded for phylogenetic analyses. The details are presented in Table 1. Sequences were aligned by the online version of the multiple sequence alignment program MAFFT v7 (Katoh and Standley 2013) and were manually adjusted in BIOEDIT v7.1.3.0 (Hall 1999). Phylogenetic analyses were conducted using Bayesian inference (BI) in MRBAYES v3.2.6 (Ronquist and Huelsenbeck 2003) and maximum likelihood (ML) in IQTREE v1.5.6 (Nguyen et al. 2014). For BI analyses, the best model was selected by AIC in MRMODELTEST 2.3, and gaps were treated as missing data (Nylander 2004; Örstadius et al. 2015). Four Markov chains (MCMCs) were run for two million generations, with sampling every 100^th^ generation. The first 25% of trees were discarded (Ronquist and Huelsenbeck 2003). ML analyses were executed by applying the ultrafast bootstrap approximation with 1000 replicates. The sequence alignment was deposited in TreeBASE (http://purl.org/phylo/treebase/phylows/study/TB2:S27605?x-access-code=ad75ae6bd4198cfa6d444a895863bc1b&format=html).

**Table 1.** Sequences used in this study. Newly generated sequences are in bold.

Taxon	Voucher	Locality	ITS	LSU	*tef-1*α	β-*Tub*
*Coprinellus christianopolitanus*	LO141-08 type	Sweden	KC992944	KC992944	KJ732823	–
*C. disseminatus*	SZMC-NL-2337		FM878017	FM876274	–	FN396282
*C. silvaticus*	LÖ172-08	Sweden	KC992943	KC992943	KJ732822	KJ664911
*C. truncorum*	SZMC-NL-1101	Sweden	FM878006	FM876262	FM897225	FN396328
*Cystoagaricus hirtosquamulosus*	Ramsholm800927	Finland	KC992945	KC992945	–	–
*C. olivaceogriseus*	WK 8/15/63-5 (MICH) Type	USA	KC992948	KC992948	–	–
*C. sylvestris*	LÖ191-92	Sweden	KC992949	KC992949	–	–
*C. squarrosiceps*	Laessoe44835	Ecuador	KC992950	–	–	–
*C. strobilomyces*	E. Nagasawa 9740		AY176347	AY176348	–	–
*Heteropsathyrella macrocystidia*	HMJAU37802 Type	China:Jilin	**MW405102**	**MW413359**	**MW411004**	**MW410997**
*H. macrocystidia*	HMJAU37803	China:Jilin	**MW405101**	**MW413358**	**MW411003**	–
*H. macrocystidia*	HMJAU37912	China:Jilin	**MW405103**	**MW413360**	**MW411005**	–
*Kauffmania larga*	LAS97-054	Sweden	DQ389695	DQ389695	–	–
*K. larga*	LÖ223-90	Sweden	DQ389694	DQ389694	KJ732824	KJ664912
*Psathyrella abieticola*	Smith58673 (MICH) Type	USA	KC992891	KC992891	–	–
*P. amygdalinospora*	HMJAU37952 Type	China:Sichuan	**MW405104**	**MW413361**	**MW410999**	**MW410991**
*P. amygdalinospora*	HMJAU57044	China:Sichuan	**MW405105**	–	–	–
*P. conferta*	GE02.007 (PC) Type	France	KC992890	KC992890	–	–
*P. echinata*	ZT12073	NewZealand	KC992925	KC992925	–	KJ664900
*P. fagetophila*	LÖ210-85 (M) Type	Sweden	KC992902	KC992902	–	KJ664879
*P. fennoscandica*	HMJAU37918	China:Heilongjiang	MG734723	**MW413365**	**MW411000**	**MW410993**
*P. fennoscandica*	LÖ484-05 Type	Sweden	KC992903	KC992903	KJ732790	KJ664881
*P. fennoscandica*	LÖ95-96	Sweden	KC992904	KC992904	KJ732791	KJ664882
*P. fusca*	LÖ287-04	Sweden	KC992892	KC992892	KJ732779	–
*P. mucrocystis*	LÖ103-98	Sweden	DQ389700	–	KJ732810	KJ664901
*P. noli-tangere*	LÖ83-03 Neotype	Sweden	DQ389713	DQ389713	–	KJ664890
*P. oboensis*	HMJAU37936	China:Yunnan	MT429164	**MW413366**	–	**MW410996**
*P. oboensis*	DED 8234 Type	SãoTomé	NR148107	–	–	–
*P. olympiana*	LÖ32-02	Sweden	DQ389722	DQ389722	KJ732817	KJ664906
*P. panaeoloides*	LÖ44-03	Sweden	DQ389719	DQ389719	KJ732782	KJ664873
*P. panaeoloides*	HMJAU23696	China:Jilin	MG734733	MH155958	–	MH161165
*P. pertinax*	HMJAU6830	China:Jilin	MG734735	–	–	**MW410995**
*P. pertinax*	LO259-91 Neotype	Sweden	DQ389701	DQ389701	KJ732809	–
*P. piluliformis*	HMJAU37922	China:Heilongjiang	MG734716	**MW413364**	**MW411001**	**MW410994**
*P. piluliformis*	LÖ162-02	Germany	DQ389699	DQ389699	KJ732808	KJ664899
*P. piluliformoides*	HMJAU37923 Type	China:Yunnan	**MW405106**	**MW413362**	**MW411002**	–
*P. pygmaea*	LÖ97-04	Sweden	DQ389718	DQ389718	KJ732811	KJ664902
*P. pygmaea*	HMJAU37850	China:Jilin	MG734744	MH155959	MH161170	MH161166
*P. rybergii*	LÖ373-06 Type	Sweden	KC992893	KC992893	KJ732781	KJ664872
*P. saponacea*	HMJUA 37935	China:Shanxi	MH155965	MH155960	–	MH161167
*P. saponacea*	LÖ204-96	Sweden	DQ389717	–	KJ732780	KJ664871
*P. seminuda*	Smith34091 (MICH) Type	USA	KC992907	KC992907	–	–
*P. senex*	HMJAU4450	China:InnerMongolia	MG734732	–	–	**MW410992**
*P. senex*	LÖ115-02	Germany	DQ389712	DQ389712	–	KJ664880
*P. truncatisporoides*	HMJAU37947 Type	China:Zhejiang	**MW405107**	**MW413363**	**MW410998**	**MW410990**
*P. truncatisporoides*	HMJAU57045	China:Zhejiang	**MW405108**	–	–	–
*P. warrenensis*	Smith70162 (MICH) Type	USA	KC992906	KC992906	–	–
*Typhrasa gossypina*	Schumacher024	Germany	KC992946	KC992946	KJ732825	–
*T. nanispora*	Barta980706 Type	Austria	KC992947	KC992947	–	–
Outgroup						
*Coprinopsis cineraria*	CBM-FB-24142 Type	Japan	KC992962	–	–	–
*C. musae*	JV06-179 Type	Denmark	KC992965	KC992965	–	KJ664920
*C. submicrospora*	AH27055 Type	Spain	KC992959	KC992959	–	KJ664918
*C. udicola*	AM1240 Type	Germany	KC992967	KC992967	KJ732831	KJ664922
*C. uliginicola*	Smith34903 (MICH) Type	USA	KC992960	KC992960	–	–

## Results

### Phylogenetic results

The aligned complete dataset consisted of 54 taxa and 2606 characters (ITS 711 bp, LSU 829 bp, Tef 1^st^ 69 bp, Tef 2^nd^ 136 bp, Tef 3^rd^ 497 bp, Tub 1^st^ 125 bp, and Tub 2^nd^ 239 bp). Due to the different number of models supported by Mrbayes and IQtree, the best models are calculated separately, and the results are as follows: the best models for Bayesian analysis were GTR+I+G for the ITS, LSU, Tef 3^rd^, and Tub 2^nd^, HKY+I for Tef 1^st^, SYM+G for Tef 2^nd^, and SYM+I+G for Tub 1^st^; the best models for ML analysis were TIM2+F+I+G4 for the ITS and LSU, TNe+FQ+I for Tef 1^st^ and Tef 2^nd^, TIM2+F+G4 for Tef 3^rd^, TIMe+FQ+G4 for Tub 1^st^, and HKY+F+G4 for Tub 2^nd^.

**Figure 1.**
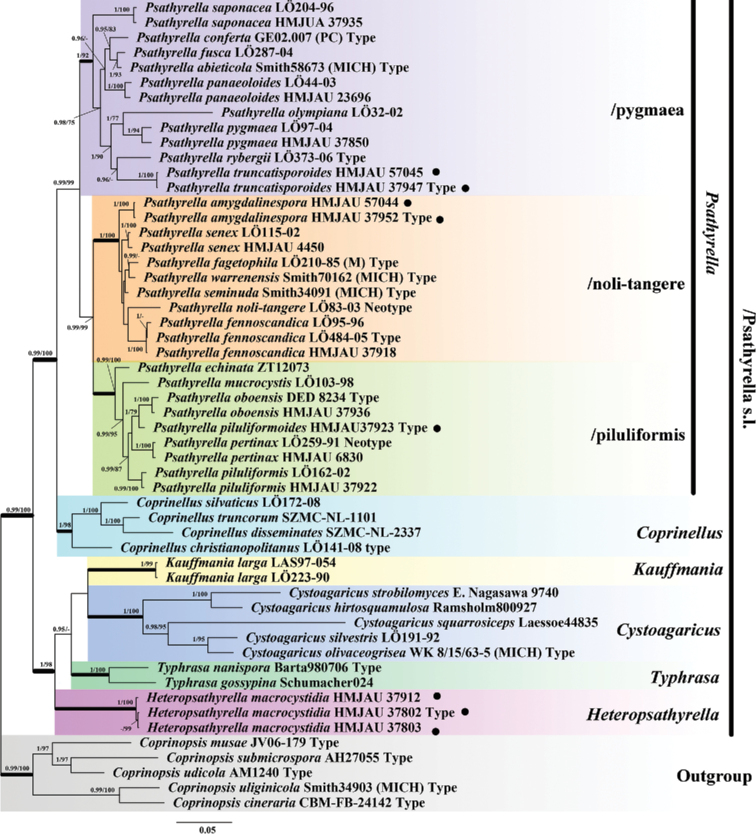
Phylogram generated by Bayesian inference (BI) analysis based on sequences of a concatenated data set from four nuclear genes (ITS, LSU, *tef-1α* and *β-tub*), rooted with *Coprinopsis* spp. Bayesian inference (BI-PP) ≥ 0.95 and ML bootstrap proportions (ML-BP) ≥ 75 are shown as BI-PP/ML-BP. ● indicates newly described taxa.

For Bayes analysis, the average standard deviation of split frequencies less than 0.01 after 610 thousand generations. The Bayesian inference (BI) and ML bootstrap proportions are shown in the Bayesian tree (Fig. 1). In addition, the ML tree is shown in Suppl. material 2: Figure S2. The phylogenetic tree analyses recovered 8 major supported clades (6 genura), with a high statistical support value (BPP ≥ 0.95, bootstrap ≥ 75). They are *Psathyrella* (including 3 clades), *Coprinellus*, *Kauffmania*, *Cystoagaricus*, *Typhrasa*, and the new genus – *Heteropsathyrella*. *P. amygdalinospora*, *P. piluliformoides*, *P. truncatisporoides* were separated into individual lineages and are independent from the close taxa in *Psathyrella*. *P. amygdalinospora* forms a distinct lineage in the /pygmaea clade, *P. piluliformoides* belongs to /piluliformis and groups together with *P. oboensis* Desjardin & B.A. Perry, and *P. truncatisporoides* belongs to /noli-tangere and groups together with *P. rybergii* Örstadius & E. Larss. *H. macrocystidia* forms a distinct lineage and groups together with the lineage consisting of *Cystoagaricus*, *Kauffmania*, and *Typhrasa*.

### Taxonomy

#### Heteropsathyrella

Taxon classificationFungiAgaricalesPsathyrellaceae

T. Bau & J.Q. Yan gen. nov.

EB98B8C2-4005-5AFB-9317-18E7E1A1776C

838372

##### Remarks.

Pileus hygrophanous, tawny to brown, non-deliquescent. Veil present. Lamellae adnexed. Stipe central, hollow. Basidiospores ellipsoid to subellipsoid, smooth, brown in 5% KOH, pale mouse grey in H_2_SO_4_. Hymenium hyaline. Basidia monomorphic. Pseudoparaphyses abundant and regularly distributed. Pleurocystidia and cheilocystidia present. Pileipellis composed of saccate to subglobose cells covered by a 1 cell deep layer of periclinal hyphae which are covered by scattered and irregular deposits dissolving in 5% KOH.

##### Etymology.

*Heteropsathyrella*, referring to its morphological similarity to *Psathyrella*.

##### Type species.

*Heteropsathyrella macrocystidia* T. Bau & J.Q. Yan, *sp. nov*.

#### Heteropsathyrella macrocystidia

Taxon classificationFungiAgaricalesPsathyrellaceae

T. Bau & J.Q. Yan sp. nov.

EF9D5071-2E6C-5F9C-B45C-33F973F7C8A8

838373

Figs 2a–c , 3

##### Etymology.

*macrocystidia*, referring to its large pleurocystidia, which are up to 83 μm long.

##### Type.

China. Changbai Mountain, Antu County, Yanbian Korean Autonomous Prefecture, Jun-Qing Yan, Herbarium of Mycology, Jilin Agricultural University (HMJAU37802).

##### Diagnosis.

Differs from *Psathyrella epimyces* by saprophytic and abundant pseudoparaphyses.

Pileus 35–70 mm broad, obtusely conic when young, expanding to plane, with a small obtuse umbo, hygrophanous, tawny to brown (7C6–7D7), darker at center (7E7), striate up to 2/3 from margin, becoming dirty white as pileus dries (7A1–7B2). Veil scattered, small, white (7A1), fibrillose, evanescent. Context hygrophanous, thin and fragile, approximately 1.0–1.5 mm at the centre. Lamellae 3.0–6.0 mm broad, crowded, adnexed, dirty white (7A1–7B2), becoming pale brown to brown (7E7–7F7) as spores mature, edge white (7A1) and even. Stipe 35–100 mm long, 5.0–15 mm thick, white (7A1), cylindrical, gradually thickening towards base, fragile, hollow, but context thick, surface uneven, with small grainy bulb, covered with small, white, evanescent fibrils. Odour and taste indistinctive. Spore print grey brown (7E3–7E4).

**Figure 2.**
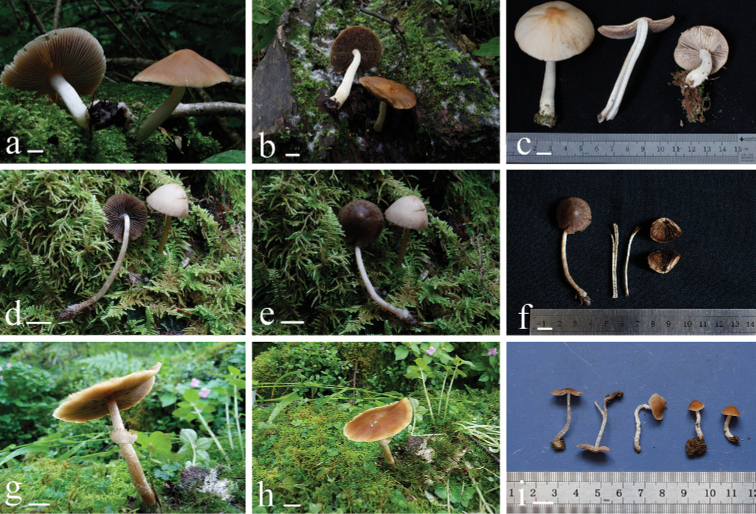
Basidiomata **a–c***Heteropsathyrella macrocystidia* (HMJAU37802) **d–f***Psathyrella amygdalinospora* (HMJAU37952) **g, h***P. piluliformoides* (HMJAU37923) **i***P. truncatisporoides* (HMJAU37947). Scale bars: 10 mm (**a–i)**.

Spores 7.8–9.2 × 4.9–5.4 μm, Q = 1.6–1.8, elongated-ellipsoid in face view, in profile flattened on one side, pale brown in water, darker brown in 5% KOH, smooth, with or without 1–2 guttules, germ pore indistinct, approximately 1.0 μm in diam. Basidia 26–34 × 7.3–9.8 μm, clavate, hyaline, 4- or 2-spored. Pseudoparaphyses abundant and regular distribution. Pleurocystidia 59–83 × 12–20 μm, abundant, utriform with broadly obtuse apex, slightly thick-walled, glabrous or covered by irregular deposits, base tapering to a long stipe. Cheilocystidia 37–56 × 9.8–17 μm, utriform to fusoid with obtuse apex, base tapering to a short stipe. Caulocystidia 29–61 × 12–22 μm, caespitose, various, utriform, fusoid or utriform with abrupt narrow neck terminating in a capitellum, base tapering to a long or short stipe. Trama of gills irregular. Pileipellis a 1–2-cell-deep layer of vesiculose cells, up to 61 μm long, covered by a 1-cell-deep layer of periclinal hyphae which are approximately 3.6 μm in diam and covered by scattered and irregular deposition dissolving in 5% KOH. Clamps present.

**Figure 3.**
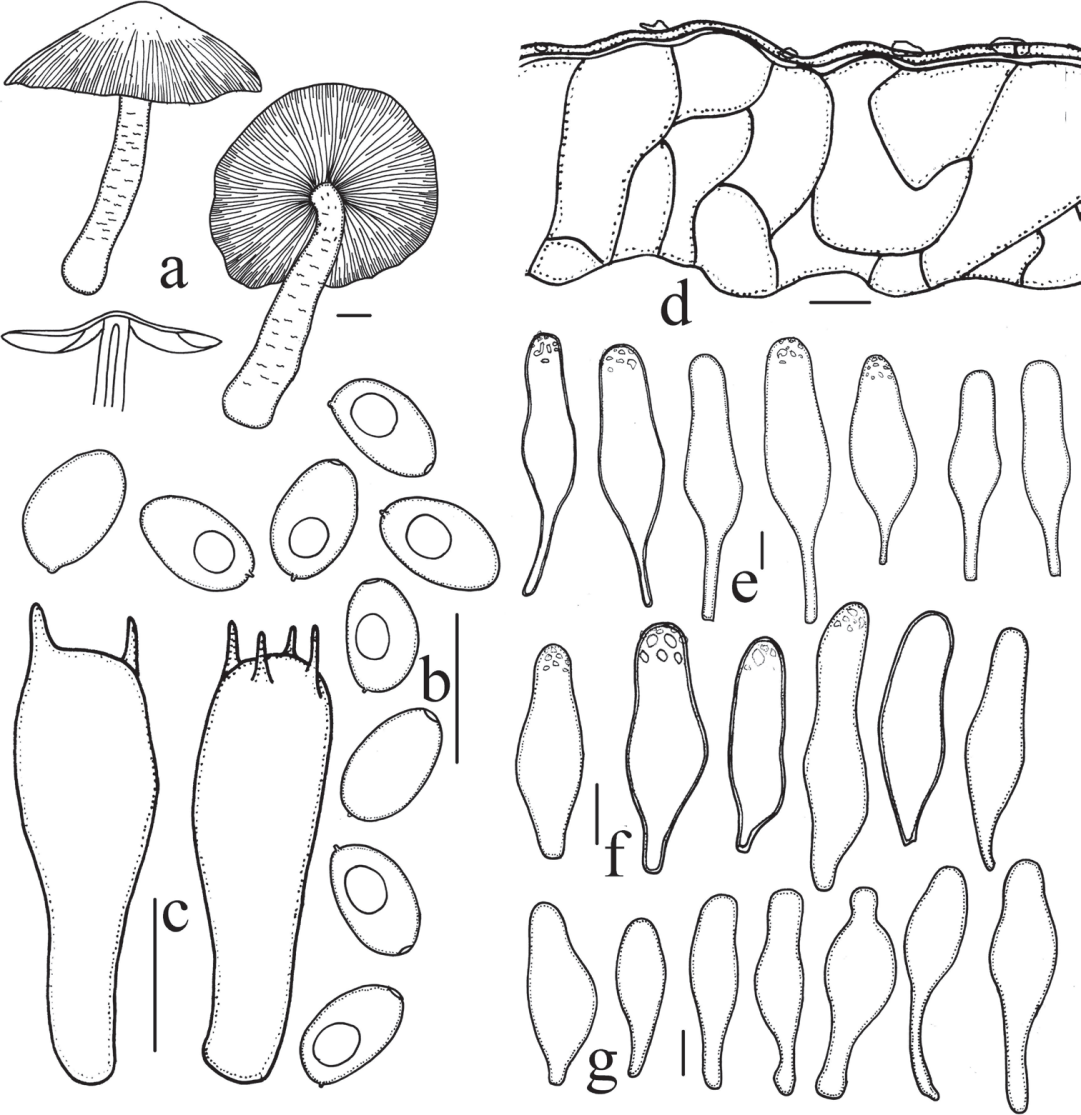
*Heteropsathyrella macrocystidia* (HMJAU37802) **a** basidiomata **b** basidiospores **c** basidia **d** pileipellis **e** pleurocystidia **f** cheilocystidia **g** caulocystidia. Scale bars: = 10 mm (**a**); 10 μm (**b–g**).

##### Habit and habitat.

Scattered on mossy rotten wood in mixed forests of larch and birch.

##### Other specimens examined.

China. Changbai Mountain, Antu County, Yanbian Korean Autonomous Prefecture, Jun-Qing Yan, 16 July 2016, HMJAU37803; 28 July 2017, HMJAU37912.

#### Psathyrella amygdalinospora

Taxon classificationFungiAgaricalesPsathyrellaceae

T. Bau & J.Q. Yan sp. nov.

DDDA33DB-A3AE-59C8-8545-1E27F30A23E4

838374

Figs 2d–f , 4

##### Etymology.

Referring to the spore shape.

##### Type.

China. Scenic Spot of Kangding Love Song (Mugecuo), Kangding City, Tibetan Autonomous Prefecture of Garzê, Sichuan Province, 30°08'49.19"N, 101°51'39.18"E, 3790 m, 21 August 2017, Jun-Qing Yan, Herbarium of Mycology, Jilin Agricultural University (HMJAU37952).

##### Diagnosis.

Differs from *P. obtusata* by its spores, ovoid in front view, amygdaliform in profile and dark brown and gradually becoming black-brown in 5% KOH.

Pileus 15–25 mm broad, paraboloid, hygrophanous, chestnut (8F6–8F7), becoming dirty white (8A1–8B1) as pileus dries. Veil not observed. Context approximately 2.0 mm at the centre, fragile, concolorous with pileus. Lamellae 4.0 mm, light brown (8D3–8D5), edges white (8A1), even. Stipes 45–60 mm long, 2.5–3.0 mm thick, fragile, hollow, cylindrical, equal or slightly expanded at base, dirty white (8A1–8B1). Odour and taste indistinctive.

Spores 8.8–9.7 × 4.9–5.8 μm, Q = 1.5–1.9, ovoid in front view, amygdaliform in profile, reddish brown in water, dark brown and gradually becoming black-brown in 5% KOH, inamyloid, smooth, germ pore absent. Basidia 17–20 × 7.3–9.8 μm, clavate, hyaline, 4-spored. Pleurocystidia abundant, 44–68 × 9.8–13 μm, fusiform to narrowly utriform, thin-walled, apex obtuse to subacute, rarely subcapitate. Pleurocystidioid cheilocystidia abundant, 22–32 × 7.3–12 μm, fusiform to utriform, short mucronate or obtuse at apex, rarely mixed with pyriform cells. Trama of gills irregular. Pileipellis consisting of a 1–2-cell-deep layer of subglobose cells that were 30–40 μm broad. Clamps present.

**Figure 4.**
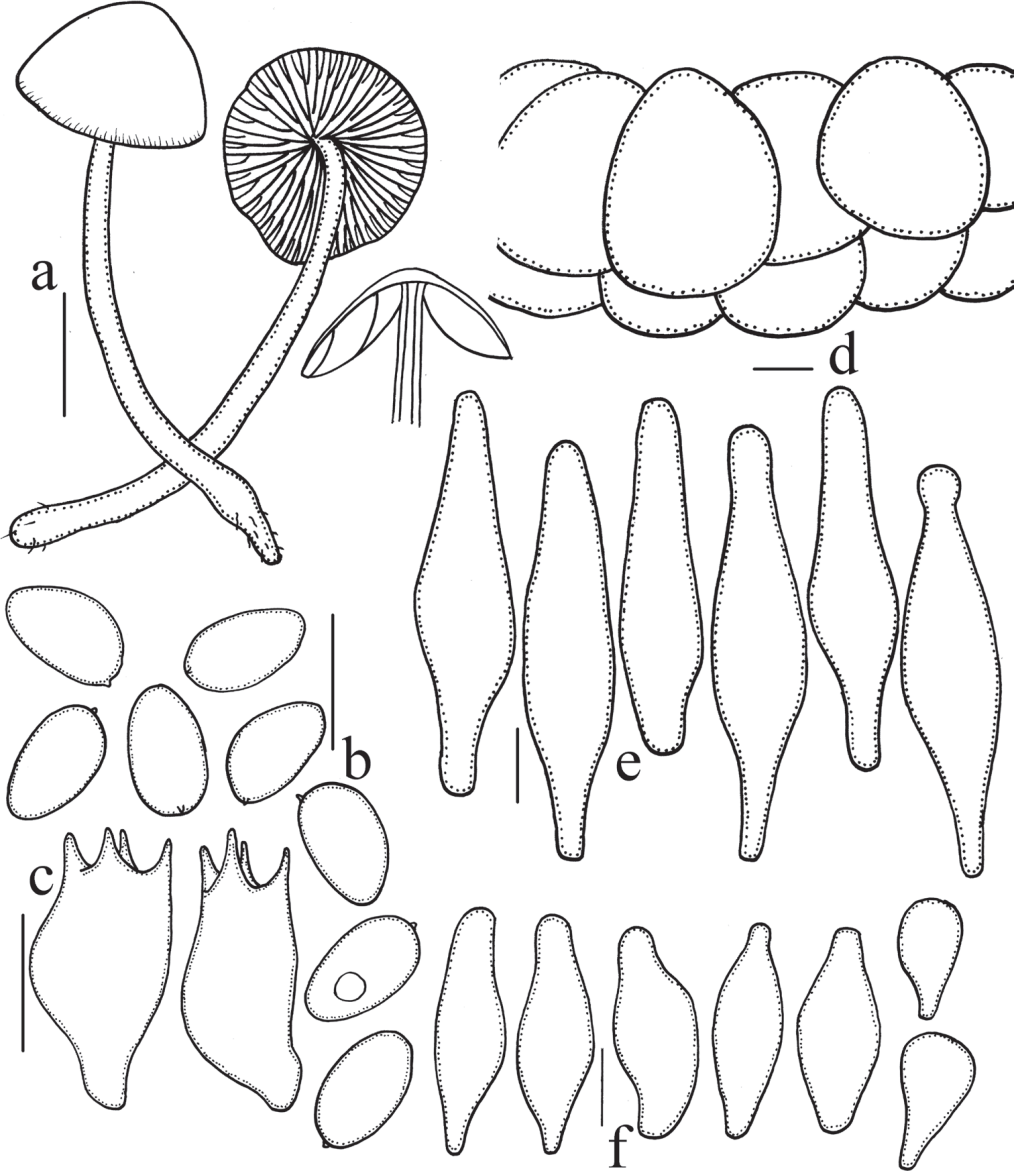
*Psathyrella amygdalinospora* (HMJAU37952) **a** basidiomata **b** basidiospores **c** basidia **d** pileipellis **e** pleurocystidia **f** cheilocystidia. Scale bars: 10 mm (**a**); 10 μm (**b–f**).

##### Habit and habitat.

Scattered on mosses in mixed forests of *Cunninghamia* spp., *Pinus* spp. and *Quercus semecarpifolia*.

##### Other specimens examined.

China. Scenic Spot of Kangding Love Song (Mugecuo), Kangding City, Tibetan Autonomous Prefecture of Garzê, Sichuan Province, 22 August 2017, Jun-Qing Yan, HMJAU57044.

#### Psathyrella piluliformoides

Taxon classificationFungiAgaricalesPsathyrellaceae

T. Bau & J.Q. Yan sp. nov.

2D0F060E-2537-5CBB-B6E4-E9EC0A5FB97E

838375

Figs 2g, h , 5

##### Etymology.

Reference to its characteristics similar to *Psathyrella piluliformis*.

##### Type.

China. Kunming Institute of Botany, Kunming City, Yunnan Province, 9 September 2017, Herbarium of Mycology, Jilin Agricultural University (HMJAU37923).

##### Diagnosis.

Differs from *Psathyrella piluliformis* by having ring and yellow amorphous incrustation at the apex of pleurocystidia.

Pileus 50–60 mm broad, plane, hygrophanous, brown (7C7–7D7) at centre, pale (6B6–6B7) at margin, smooth, striations indistinct at margin. Context thin and fragile, approximately 2.0 mm at the centre, same colour as pileus. Lamellae approximately 4.0 mm, very closed, pale coffee (6C5–6D5), edges paler and even (6B4). Stipe 5.5 mm long, 5.0 mm thick, fragile, cylindrical, hollow, slightly thickened towards base, white (6A1) at apex, base slightly brown, with white evanescent squama. Ring present at 1/3 from stipe apex.

Spores 5.6–6.3 × 3.1–4.4 μm, Q = 1.3–1.9, ellipsoid to oblong-ellipsoid, in profile flattened on one side, pale brown in water, dirty brown in 5% KOH, inamyloid, smooth, germ pore distinct, truncate, 1.1–1.9 μm broad. Basidia 15–18 × 4.9–6.1 μm, clavate, hyaline, 4- or 2-spored. Pleurocystidia 39–54 × 11–15 μm, abundant, utriform to narrowly utriform, or lageniform, rarely fusiform, thick-walled or thin-walled, apex obtuse or broadly obtuse, covered by yellow amorphous incrustation, dissolving in 5% KOH. Cheilocystidia scattered, 24–37 × 9.8–15 μm, utriform, thick-walled or thin-walled, apex obtuse or broadly obtuse, mixed with subglobose to spheropedunculate cells, cells 11–16 × 9.8–14 μm, slightly thick-walled or not. Trama of gills irregular. Pileipellis consisting of a 2–3-cell-deep layer of subglobose cells 34–40 μm broad. Clamps present.

**Figure 5.**
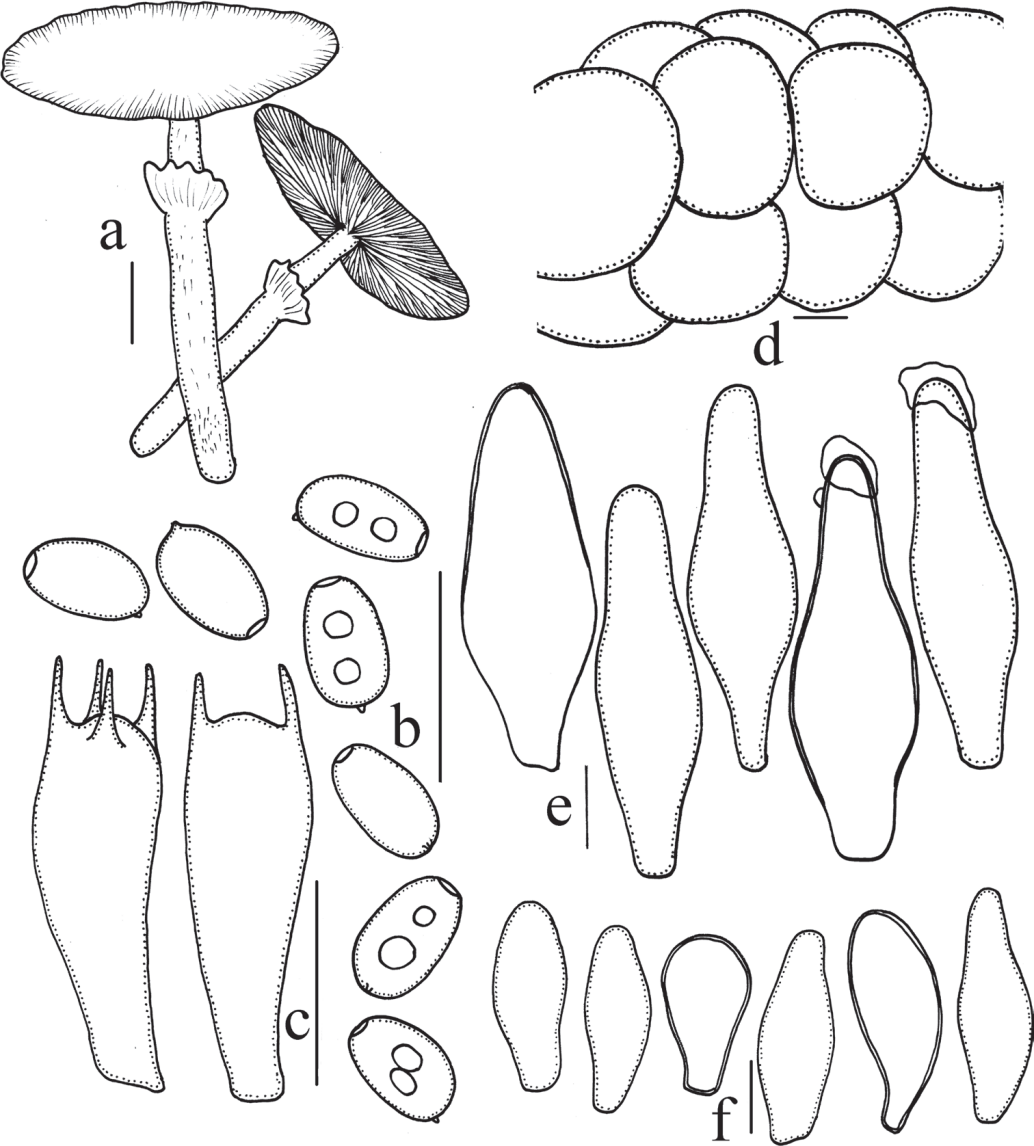
*Psathyrella piluliformoides* (HMJAU37923) **a** basidiomata **b** basidiospores **c** basidia **d** pileipellis **e** pleurocystidia **f** cheilocystidia. Scale bars: 10 mm (**a**); 10 μm (**b–f**).

##### Habit and habitat.

Solitary on moss.

#### Psathyrella truncatisporoides

Taxon classificationFungiAgaricalesPsathyrellaceae

T. Bau & J.Q. Yan sp. nov.

0A61C127-9AD4-5AD4-B115-13EDC0545B7E

838378

Figs 2i , 6

##### Etymology.

Referring to the truncate spore.

##### Type.

China. Wulingken, Baishanzhu, Qingyuan County, Lishui City, Zhejiang Province, Tolgor Bau, Jun-Qing Yan, 16 August 2015, Herbarium of Mycology, Jilin Agricultural University (HMJAU37947).

##### Diagnosis.

Differs from *P. rybergii* by its shorter spores (6.8–7.8 μm).

Pileus 8.0–13 mm broad, spreading broadly conical to plane, hygrophanous, pale brown (7C6–7D7), white (7A1–7B1) at margin, striate up to 2/3 from margin. Veil of a thin coating of white to dirty white (7A1–7B1) fibrils, evanescent. Context thin and very fragile, same colour as pileus, approximately 1.0 mm at centre. Lamellae approximately 1.5 mm broad, pale brown (7B4–7C4), close, adnate, margin even. Stipes 10–25 mm long, approximately 1.5 mm thick, white (7A1), fragile, hollow, smooth but irregularly lumpy, with the base slightly expanding or not. Odour and taste indistinctive.

Spores (5.8)6.8–7.8(8.3) × 4.4–4.9 μm, Q=1.2–1.8, broadly ellipsoid to ellipsoid, in profile flattened on one side, inamyloid, smooth, apex obviously truncate, germ pore distinct, 1.5–2.4 μm broad. Basidia 13–17 × 6.1–7.3 μm, clavate, 4-spored. Pleurocystidia 37–49 × 12–16 μm, utriform to broadly utriform, with obtuse to broad apex, base tapering to a long or short stipe, thin-walled. Cheilocystidia 19–31 × 7.3–12 μm, abundant, similar to pleurocystidia, rarely spheropedunculate, rarely with crystals. Trama of gills irregular. Pileipellis a hymeniderm with 29–39 μm broad cells. Clamps present.

**Figure 6.**
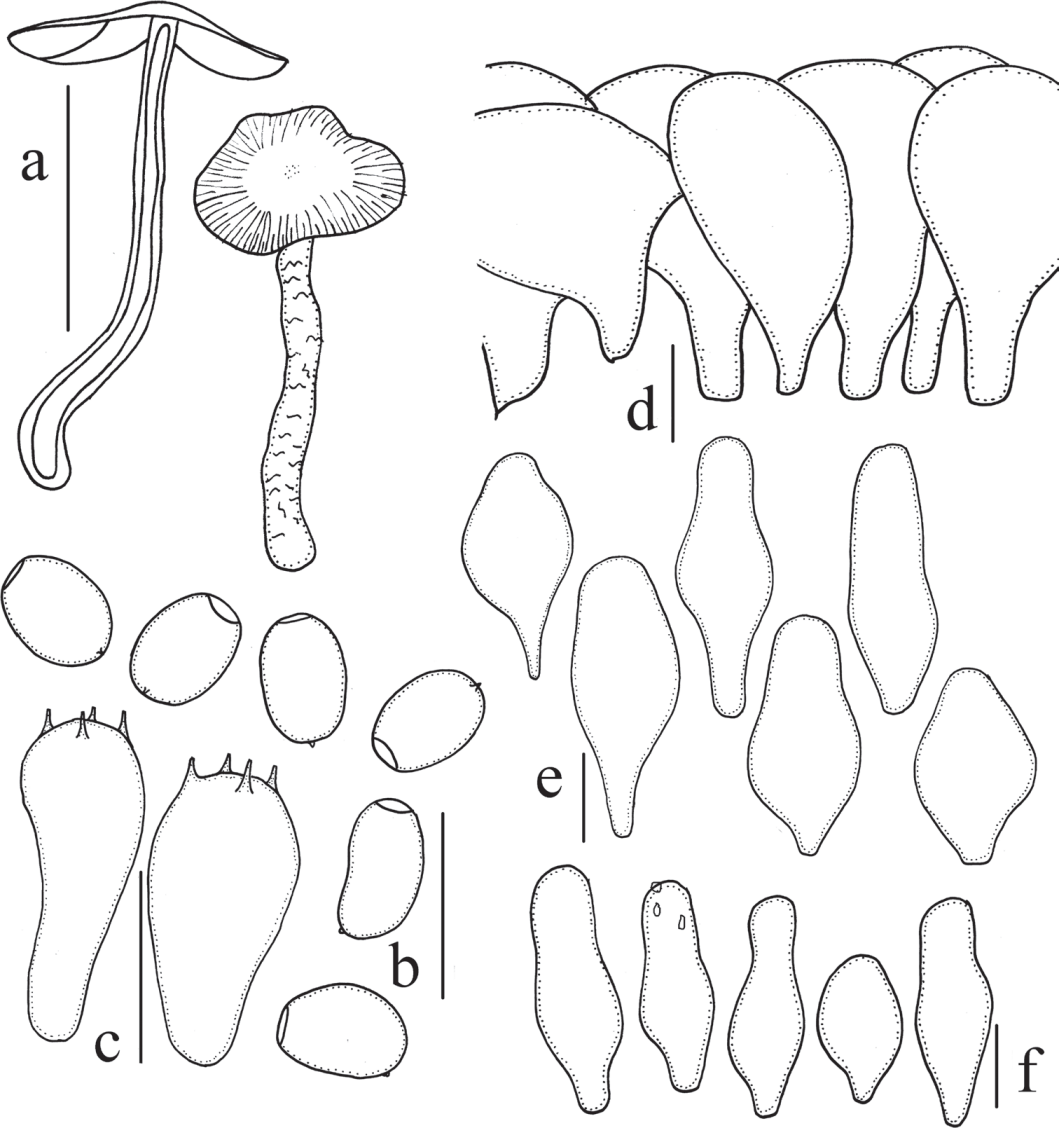
*Psathyrella truncatisporoides* (HMJAU37947) **a** basidiomata **b** basidiospores **c** basidia **d** pileipellis **e** pleurocystidia **f** cheilocystidia. Scale bars: 10 mm (**a**); 10 μm (**b–f**).

##### Habit and habitat.

Scattered, terrestrial, in bamboo forest.

##### Other specimens examined.

China. Wulingken, Baishanzhu, Qingyuan County, Lishui City, Zhejiang Province, Tolgor Bau, Jun-Qing Yan, 18 August 2015, HMJAU57045.

## Discussion

The species in the family Psathyrellaceae can be roughly divided into two types by macromorphology: psathyrelloid and coprinoid. *Heteropsathyrella* is macromorphologically similar to *Psathyrella* s.s. but phylogenetically and micromorphologically distinguished from it, differing in the special pileipellis which composed of utriform to subglobose cells covered by a 1 cell deep layer of periclinal hyphae and abundant pseudoparaphyses. There are no other genera in this family, like *Heteropsathyrella*, that match the characteristics of psathyrelloid basidiomata, lamellae adnexed, basidia monomorphic, pseudoparaphyses abundant and pileipellis composed of a cellular subpellis below a hyphal suprapellis covered by scattered and irregular deposits, which dissolve in 5% KOH. Based on the study of this family (Smith 1972; Kits van Waveren 1985; Nagy et al. 2013; Örstadius et al. 2015), a detailed feature comparison between *Heteropsathyrella* and related genera are shown in Table 2. The type species, *H. macrocystidia*, is characterized by stout basidiomata, large pleurocystidia up to 80 μm long, and the generic characters above cited. Thus, this taxon is obviously unique and distinguished from all known species. In the case of not comparing the pileipellis and pseudoparaphyses, few species have the aspect of *H. macrocystidia*: *P. epimyces* (Peck) A.H. Sm. has stout basidiomata, and large pleurocystidia up to 70 μm long, but parasitic on *Coprinus*- or *Coprinopsis*- species (Smith 1972); *P. parvifibrillosa* A.H. Sm. and *P. lauricola* A.H. Sm. & Hesler has large pleurocystidia up to 70 μm long, but basidiomata small, and the shape of pleurocystidia are fusoid and utriform without long stipe at the base, respectively (Smith 1972).

**Table 2.** A summary of morphological characteristics used to discriminate the ten genera.

	* Coprinellus *	* Coprinopsis *	* Cystoagaricus *	* Heteropsathyrella *	* Homophron *	* Kauffmania *	* Lacrymaria *	* Parasola *	* Psathyrella *	* Typhrasa *
**Veil**	subglobose cells, hyphae, or absent	hyphae, subglobose cells, or mixtures	hyphae	**hyphae**	absent	hyphae	hyphae	absent	hyphae, rarely subglobose cells	hyphae
**Cap or lamellae**	fully, partilly, or non-deliquescent	deliquescent, rarely non-deliquescent	non-deliquescent	**non-deliquescent**	non-deliquescent	non-deliquescent	non-deliquescent	non-deliquescent, or collapsing	non-deliquescent	non-deliquescent
**Spore surface**	smooth, rarely warty	smooth, rarely warty or with myxosporium	smooth	**smooth**	smooth	smooth	often warty	smooth	smooth, rarely granulose or with myxosporium	smooth
**Basidia**	mono-, di-, tri-, or tetramorphic	Dimorphic	monomorphic	**monomorphic**	monomorphic	monomorphic	mono- to dimorphic	di- to trimorphic	monomorphic	monomorphic
**Pseudoparaphyses**	present	present, rarely absent	absent	**present**	absent	absent	absent	present	rarely present	absent
**Pileipellis**	hymeniderm to paraderm	Cutis	paraderm	**hymeniderm to paraderm, covered by a 1 cells deep layer of periclinal hyphae**	hymeniderm to paraderm	hymeniderm to paraderm	hymeniderm	hymeniderm	hymeniderm, paraderm, rarely cutis	hymeniderm to paraderm
**Pileocystidia**	often present	Absent	absent	**abundant and regular distribution**	simple hairs sometimes present	absent	absent	absent	very rarely present	absent
**Sclerocystidia**	sometimes present	Absent	absent	**absent**	absent	absent	absent	absent	absent	absent

For several of the already formally described and circumscribed clades within *Psathyrella*, phylogenetic analyses suggest that they include a morphologically heterogeneous assemblage of species, and morphological characterization is difficult (Örstadius et al. 2015). The boundaries between species in the /noli-tangere clade are difficult to characterize; these taxa share the characteristics of spores less than 10 μm long, and utriform, fusiform, lageniform or transition-type pleurocystidia present at the same time. The new species *P. amygdalinospora* forms an independent lineage and differs from the others in spores being ovoid in front view, amygdaliform in profile, and germ pore being absent. Macromorphologically, this species is similar to *P. obtusata* (Pers.) A.H. Sm. and *P. fulvescens* (Romagn.) M.M. Moser ex A.H. Sm, but the spores of *P. obtusata* are ellipsoid to oblong-ellipsoid and pale yellow in 5% KOH (Örstadius and Knudsen 2012), while *P. fulvescens* has an obvious germ pore (Smith 1972).

*P. amygdalinospora* can be classified into section Pennatae in Kits van Waveren’s classification system (Kits van Waveren 1985) and in subsection Limicolae in Smith’s classification system (Smith 1972). The closest related species can be separated as follows: the pleurocystidia of *P. pennata* (Fr.) A. Pearson & Dennis are fusoid-ventricose with an acute apex and thickened wall (Kits van Waveren 1985); the spores of *P. borealis* A.H. Sm. are ellipsoid and have an obvious germ pore (Smith 1972).

The species in the /pygmaea clade share abundant cheilocystidia and utriform pleurocystidia. The new species *P. truncatisporoides* forms a distinct lineage and groups together with *P. rybergii* Örstadius & E. Larss. in this clade. The closely related *P. rybergii* differs in having spore lengths of 8.5–9.5 μm. Macromorphologically, there are hardly any other species that match the characteristics of *P. truncatisporoides* and they can be separated as follows: *P. rubiginosa* A. H. Sm. has subdistant lamellae and a very inconspicuous germ pores (Smith 1972); the pleurocystidia of *P. noli-tangere* (Fr.) A. Pearson & Dennis are narrowly utriform to lageniform and rarely forked (Kits van Waveren 1985); the spores of *P. elliptispora* A.H. Sm. are 8.0–11.0 μm long (Smith 1972).

The morphological boundary of the /piluliformis clade is basically the same as that of section Hydrophilae delineated by Kits van Waveren (1985). The new species *P. piluliformoides* forms a distinct lineage in this clade and can be separated by having an obvious ring. The closely related *P. oboensis* also exhibits very closed lamellae but differs in absence of a ring and clavate-mucronate pleurocystidia. Few species have been described resembling *P. piluliformoides* and they can be separated as follows: *P. piluliformis* (Bull.) P.D. Orton has no ring and without yellow amorphous incrustation at the apex of pleurocystidia (Örstadius and Knudsen 2012); *P. laevissima* (Romagn.) Singer has mucronate pleurocystidia (Kits van Waveren 1985).

## Supplementary Material

XML Treatment for Heteropsathyrella

XML Treatment for Heteropsathyrella macrocystidia

XML Treatment for Psathyrella amygdalinospora

XML Treatment for Psathyrella piluliformoides

XML Treatment for Psathyrella truncatisporoides

## Supplementary materials

Supplementary material 1 Figure S1. Photographs under the microscope Data type images Explanation note *Heteropsathyrella macrocystidia*: a. Basidiospores, Basidia, Pseudoparaphyses, and Pleurocystidia; b. Marginal cell; c. Pileipellis; *Psathyrella amygdalinospora*: d. Basidiospores; e. Pleurocystidia; f. Marginal cell; *P. piluliformoides*: g. Basidiospores; h1. Apex of pleurocystidia covered by yellow amorphous incrustation in water; h2. Pleurocystidia; i. Marginal cell; *P. truncatisporoides*: j. Basidiospores; k. Pleurocystidia; l: Marginal cell. Observed under 5% aqueous KOH. Congo Red was used as a stain when necessary. Scale bars: 10 μm. https://binary.pensoft.net/file/547333This dataset is made available under the Open Database License (http://opendatacommons.org/licenses/odbl/1.0/). The Open Database License (ODbL) is a license agreement intended to allow users to freely share, modify, and use this Dataset while maintaining this same freedom for others, provided that the original source and author(s) are credited.Tolgor Bau, Jun-Qing Yan

Supplementary material 2 Figure S2. Phylogram generated by maximum likelihood (ML) analysis Data type phylogenetic Explanation note Phylogram generated by maximum likelihood (ML) analysis based on sequences of a concatenated data set from four nuclear genes (ITS, LSU, *Tef-1α* and *β-tub*) rooted with *Coprinopsis* spp. ML bootstrap proportion (ML-BP) ³ 75% is shown ● indicates newly described species.. https://binary.pensoft.net/file/547334This dataset is made available under the Open Database License (http://opendatacommons.org/licenses/odbl/1.0/). The Open Database License (ODbL) is a license agreement intended to allow users to freely share, modify, and use this Dataset while maintaining this same freedom for others, provided that the original source and author(s) are credited.Tolgor Bau, Jun-Qing Yan
